# Carapanosins A–C from Seeds of Andiroba (*Carapa guianensis*, Meliaceae) and Their Effects on LPS-Activated NO Production

**DOI:** 10.3390/molecules22030502

**Published:** 2017-03-22

**Authors:** Keiichiro Higuchi, Teppei Miyake, Shoko Ohmori, Yoshimi Tani, Katsuhiko Minoura, Takashi Kikuchi, Takeshi Yamada, Reiko Tanaka

**Affiliations:** Laboratory of Medicinal Chemistry, Osaka University of Pharmaceutical Sciences, 4-20-1 Nasahara, Takatsuki, Osaka 569-1094, Japan; rikiaifuni815@yahoo.co.jp (K.H.); teppei-727@ezweb.ne.jp (T.M.); syokentosmar0316@gmail.com (S.O.); tora-love.19920906@ezweb.ne.jp (Y.T.); minoura@gly.oups.ac.jp (K.M.); t.kikuchi@gly.oups.ac.jp (T.K.); yamada@gly.oups.ac.jp (T.Y.)

**Keywords:** *Carapa guianensis*, Meliaceae, seed oil, limonoid, Carapanosins A–C

## Abstract

Two new phragmalin-type limonoids, Carapanosins A and B (**1** and **2**), and a new gedunin-type limonoid, Carapansin C (**3**), together with five known limonoids (**4**–**8**) were isolated from the oil of *Carapa guianensis* A_UBLET_ (Meliaceae) seeds, a traditional medicine in Brazil and Latin American countries. Their structures were elucidated on the basis of spectroscopic analyses using 1D and 2D NMR techniques and HRFABMS. Compounds **1**–**8** were evaluated for their effects on the production of NO in LPS-activated mouse peritoneal macrophages. The NO inhibitory assay suggested that Compounds **3**, **6**, and **8** may be valuable as potential inhibitors of macrophage activation.

## 1. Introduction

Limonoids have mainly been found in Meliaceae and Rutaceae plants, and are modified triterpenoids that originate from a precursor with 4,4,8-trimethyl-17-furylsteroids that typically contains four highly oxidized (A, B, C, and D) rings. Meliaceae plants are distributed in tropical regions throughout the world [1]. *Carapa guianensis* AUBLET (Meliaceae) is a popular medicinal plant known as “Andiroba” in Brazil, and is in the same family as mahogany. Andiroba is a tall rainforest tree that grows up to 40 m in height. It is in the same family as mahogany and has been called Brazilian mahogany or bastard mahogany due to their similarities. The andiroba tree produces a brown, ligneous, quadrilateral nut that is approximately 3 to 4 in. in diameter and has the appearance of a chestnut. The nut from andiroba contains several oil-rich kernels and seeds that are composed of an ~60% pale yellow oil. The seed oil of andiroba was previously reported to exhibit highly efficient analgesic [2], anti-bacterial [3], anti-inflammatory [4], anti-cancerous [5], anti-tumor, anti-fungal [6], and anti-allergic properties [7] and was also found to be effective against wounds, bruises, herpes ulcers, rheumatism, ear infections, and insect bites as a repellent [8,9]. We previously reported Carapanolides A and B [10], guianolide A and B [11], Carapanolides C–I [12], Carapanolides J–L [13], Carapanolides M–S [14], and Carapanolides T–X [15] in the seed oil of andiroba. Our continuing research on the seed oil of andiroba revealed the structures of two new phragmalin-type limonoids, Carapanosins A (**1**) and B (**2**), a new gedunin-type limonoid, Carapanosin C (**3**), and five known limonoids (**4**–**8**). We herein describe the isolation and structural elucidation of the new limonoids as well as their inhibitory effects of NO production.

## 2. Results and Discussion

The oil from *C. guianensis* seeds was subjected to silica gel column chromatography, medium-pressure liquid chromatography (MPLC), and reverse phase HPLC in order to obtain the new limonoids **1**–**3** and known limonoids **4**–**8**. Known compounds were identified as Carapanolide H (**4**) [12], Swietephragmin G (**5**) [16], Swietephragmin D (**6**) [16], 17-epi-17-hydroxyazadiradione (**7**) [17], and 17-β-hydroxyazadiradion (**8**) [17] by comparisons with spectroscopic data of the literature.

**Figure 1:**
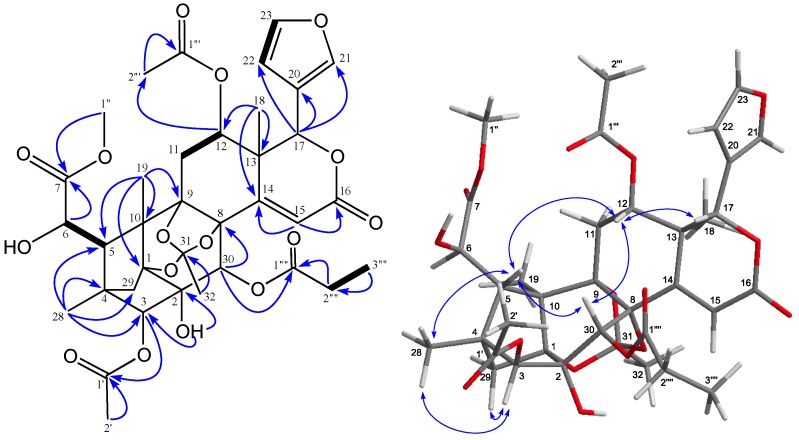
Key HMBC, COSY, and NOESY correlations for **1**.

Carapanosin A (**1**), a colorless crystal, had the molecular formula of C_36_H_42_O_16_ (*m*/*z* 731.2551 [M + H]^+^, calcd. 731.2551) as determined by HRFABMS. The IR absorption bands indicated the existence of hydroxy group (ν_max_ 3647 cm^−1^) and several carbonyl groups (1751, 1700 and 1652 cm^−1^). The UV spectrum showed a furan ring and an αβ-unsaturated δ-lactone at λ_max_ 208 nm (log ε 3.52) and 235.5 nm (log ε 3.54). ^1^H- and ^13^C-NMR spectra (Table 1) exhibited signals assignable to three tertiary methyl groups [δ_H_ 0.89, 1.34, 1.47 (each s)], two acetyl groups [δ_H_ 1.58, 2.05 (each 3 H, s); δ_C_ 20.1, 20.8 (each q), 171.1, 172.3 (each s)], a propanoyl [δ_H_ 1.10 (3 H, t), 2.31 (dq), 2.42 (m); δ_C_ 8.9 (q), 27.8 (t), 174.0 (s)], a methyl ester [δ_H_ 3.78 (3 H, s); δ_C_ 52.5 (q), 173.6 (s)], two *sp*^3^ methylenes, six *sp*^3^ methines including five oxymethines [δ_H_ 4.09 (d), 4.57 (s), 4.99 (dd), 5.29 (s), 5.71 (s); δ_C_ 68.8, 68.9, 71.4, 78.7, 83.8 (each d)], and seven *sp*^3^ quaternary carbons including four oxycarbons [δ_C_ 78.4, 83.0, 84.5, 85.2 (each s)], and the last three displacements have already been quoted above for the orthoester. I suggest seven *sp*^3^ quaternary carbons including one with a hydroxyl attached, rather than an oxygen bridge [δ_C_ 78.4], an αβ-unsaturated δ-lactone [δ_H_ 6.06 (1 H, s); δ_C_ 122.1 (d), 159.6 (s)], and a furan ring [δ_H_ 6.54 (dd), 7.42 (t), 7.55 (brs)]. In the ^1^H-^1^H COSY spectrum, cross peaks were observed between H-5–H-6, H_2_-11–H-12, H-22–H-23, and H_2_-2′′′′–H_3_-3′′′′, as shown in boldface in Figure 1.

In the HMBC spectrum (Figure 1), cross peaks were observed from H-3 [δ_H_ 4.57 (s)]/C-2 [δ_C_ 78.4 (s)], C-4, C-5, C-1’ [δ_C_ 171.1 (s)]; H-6 [δ_H_ 4.09 (d)/C-4, C-5, C-7 [δ_C_ 173.6 (s)]; H-12 [δ_H_ 4.99 (dd)]/C-11, C-13, C-14 [δ_C_ 162.7 (s)], C-17 [δ_C_ 78.7 (d)], C-18, C-1′′′ [δ_C_ 172.3 (s)]; H-15 [δ_H_ 6.06 (s)]/C-8 [δ_C_ 83.0 (s)], C-13, C-14, C-16 [δ_C_ 159.6 (s)]; H-17 [δ_H_ 5.29 (s)]/C-12 [δ_C_ 68.8 (d)], C-13, C-14, C-20 [δ_C_ 121.7 (s)], C-21 [δ_C_ 141.7 (d)], C-22 [δ_C_ 110.2 (d)]; Me-18 [δ_H_ 1.47 (s)]/C-12, C-13, C-14, C-17; Me-19 [δ_H_ 1.34 (s)]/C-1 [δ_C_ 84.5 (s)], C-5, C-9 [δ_C_ 85.2 (s)], C-10; Me-28 [δ_H_ 0.89 (s)]/C-3 [δ_C_ 83.8 (d)], C-4, C-5, C-29, H_3_-1′′ [δ_H_ 3.78 (s)]/C-7[δ_C_ 173.6 (s)]. The relative structure of **1** was determined on the basis of NOESY correlations (Figure 1). Intense NOESY correlation between H-3 and Me-28, and H-29*_pro-S_*; between H-5β and H-6, H-12, H-30β, and Me-28; between H-6 and H-30β; between H-12 and H-5β, H-17β, and H-30β; and between Me-19 and H-6, H-29*_pro-R_*, and Me-32 revealed an acetyl group at C-3 in the β orientation, C-12, a hydroxyl group at C-2, and a 2-methylpropanoyl group at C-30 in the α orientation. In addition, significant NOEs were observed between H-6 [δ_H_ 6.07 (brs)] and H-11β, H-12β and H-17β; therefore, C-6 was presumed to be in an *R*-configuration, which was consistent with Carapanolide N^14^.

**Figure 2:**
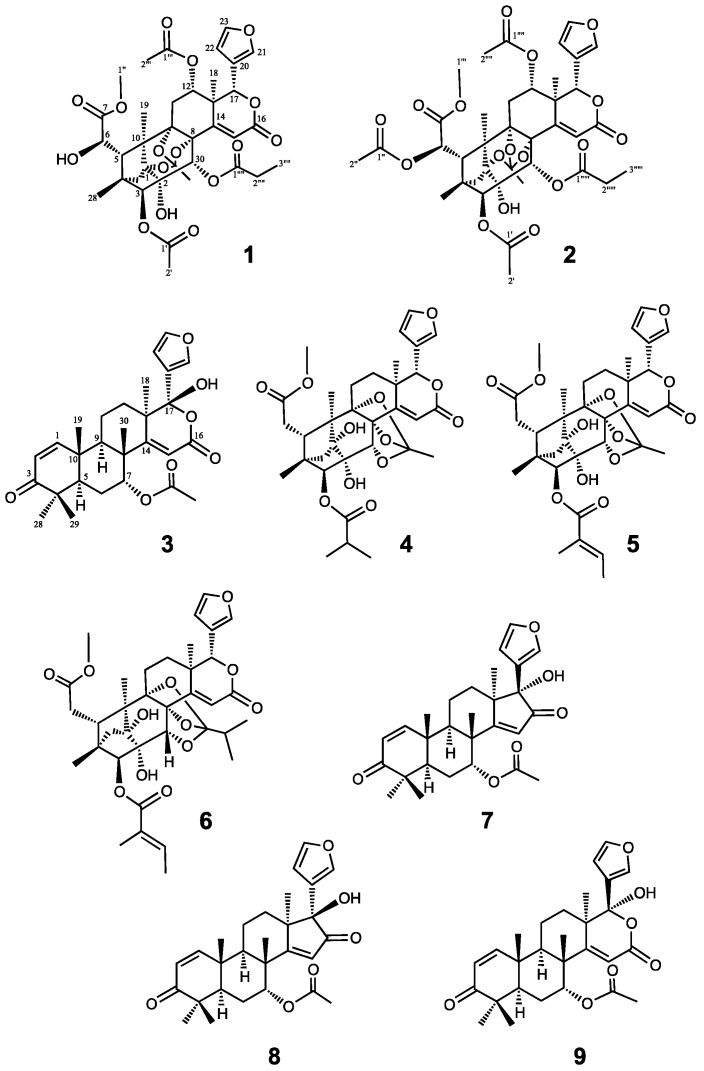
Chemical structures for Compounds **1**–**8** and nimolicinol (**9**).

Carapanosin B (**2**), a colorless amorphous, had the molecular formula of C_38_H_44_O_17_ (*m*/*z* 773.2659 [M + H]^+^, calcd. 773.2657) as determined by HRFABMS. The IR spectrum showed the presence of hydroxyl, ester groups, and an αβ-unsaturated δ-lactone at ν_max_ 3566, 1734, and 1663 cm^−1^; and the UV spectrum indicated the presence of a furan ring and an αβ-unsaturated δ-lactone at λ_max_ 213 nm (log ε 3.84) and 237.5 nm (log ε 3.62). The ^1^H- and ^13^C-NMR spectra (Table 1) displayed signals due to three tertiary methyls [δ_H_ 0.92, 1.31, 1.59 (each 3 H, s)], three acetyl groups [δ_H_ 2.08 (3 H, s), δ_C_ 169.0 (s); δ_H_ 2.20 (3 H, s), δ_C_ 171.7 (s); δ_H_ 1.55 (3 H, s), δ_C_ 170.4 (s)], a propanoyl group [δ_H_ 1.16 (3 H, t), 2.43 (1 H, dq), 2.50 (1 H, m), δ_C_ 173.9 (s)], a methyl ester [δ_H_ 3.74 (3 H, s), δ_C_ 169.2 (s)], a methylene [δ_H_ 2.00 (1 H, t), 2.35 (1 H, dd)], five *sp*^3^ methines including four oxymethines [δ_H_ 4.94 (dd), 5.19 (s), 5.91 (s), 6.31 (brd)], seven *sp*^3^ quaternary carbons including five oxycarbons [δ_C_ 83.4, 83.5, 84.1, 86.1 (each s)], an α,β-unsaturated δ-lactone [δ_H_ 6.62 (1 H, s), δ_C_ 124.2 (d), 152.6 (s), 163.4 (s)], and a furan ring [δ_H_ 6.56 (dd), 7.40 (t), 7.45 (brs)]. The ^1^H and ^13^C-NMR spectra (Table 1) of **2** were very similar to those of **1**, so **2** is estimated to be phragmalin-1,8,9-orthoacetate, except for the absence of a hydroxy group and presence of an acetyl group at C-6 [δ_H_ 6.31 (brd), δ_C_ 71.2 (d)]. In the NOESY spectrum, significant NOEs were observed between H-6 and H-11α, and Me-19, so the configuration of H-6 was determined to have the same *R* as Compound **1** and Carapanolide N [14], and its relative structure was established, as shown in Figure 2.

**Figure 3:**
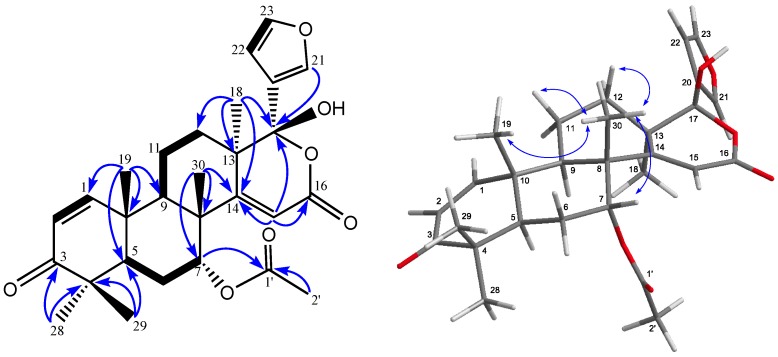
Key HMBC, COSY and NOESY correlations of Carapanosin C (**3**).

Carapanosin C (**3**) was obtained as a colorless crystal, m.p. 236–239 °C. Its molecular formula was determined to be C_28_H_34_O_7_ (*m*/*z* 483.2388 [M + H]^+^, calcd. 483.2383). The IR absorption bands indicated the existence of a hydroxy, an ester, an α,β-unsaturated six-membered ring ketone, and α,β-unsaturated δ-lactone at ν_max_ 3566, 1734, 1699, 1668 cm^−1^, and the UV absorption band indicated a λ_max_ 238.5 nm (log ε 3.74). ^1^H- and ^13^C-NMR spectra (Table 2) revealed the presence of five methyls [δ_H_ 1.08, 1.09, 1.16, 1.25, 1.36 (each 3 H, s)], a secondary acetoxy group [δ_H_ 1.98 (3 H, s), 5.25 (t); δ_C_ 169.6 (s)], αβ-unsaturated six-membered ring ketone [δ_H_ 5.87 and 7.06 (each 1 H, d), δ_C_ 203.8 (s)], an αβ-unsaturated δ-lactone [δ_H_ 5.64 (1 H, s), δ_C_ 111.0 (d), 163.4 (s), 170.3 (s)], an acetal carbon [δ_C_ 104.0 (s)] [16], and a β-substituted furan ring [δ_H_ 6.48 (dd), 7.43 (t), 7.58 (brs)], suggesting a gedunin-type limonoid. In the HMBC spectrum, the following correlations were observed: Me-18 [δ_H_ 1.16 (s)]/C-12, C-13, C-14 [δ_C_ 170.3 (s)], and C-17 [δ_C_ 104.0 (s)]; Me-19 [δ_H_ 1.25 (s)]/C-1 [δ_C_ 156.4 (d)], C-5, C-9, and C-10; Me-30 [δ_H_ 1.36 (s)]/C-7 [δ_C_ 73.2 (d)], C-8, C-9, and C-14 [δ_C_ 170.3 (s)]. The ^1^H-^1^H COSY spectrum (H-1–H-2; H-5–H_2_-6–H-7; H-9–H_2_-11–H_2_-12; H-22–H-23) revealed the positions of substituents (Figure 3). These results suggested the planer structure of **3** shown in Figure 2. Siddiqui et al. isolated nimolicinol (**9**) (m.p. 270–274 °C) (17α-hydroxy-14,15-deoxy-17-epi-gedunin) from the fruits of *Azadirachta indica* A. Juss (Neem) [18,19]. These findings suggest that the planer structure of **3** was as the same as that of **9**. However, major differences were detected in the ^1^H- and ^13^C-NMR spectra between **3** and **9**. These differences between **3** and **9** were particularly prominent in C-12 (δ_C_ 23.2 in **3**; δ_C_ 37.2 in **9**), C-9 (δ_C_ 37.2 in **3**; δ_C_ 45.5 in **9**), and C-22 (δ_C_ 125.0 in **3**: δ_C_ 110.1 in **9**), and slight differences were observed in C-5 (δ_C_ 43.5 in **3**: δ_C_ 40.5 in **9**), C-6 (δ_C_ 23.0 in **3**: δ_C_ 25.0 in **9**), C-10 (δ_C_ 40.4 in **3**: δ_C_ 42.1 in **9**), and C-13 (δ_C_ 42.0 in **3**: δ_C_ 44.5 in **9**). The relative configuration of **3** was mainly established by a NOESY experiment (Figure 3). Cross-peaks were observed Me-30/H-7β [δ_H_ 5.25 (t)], H-15, and Me-19; H-21/H-12α, H-12β, and Me-18; and Me-18/H-9α, H-12α, H-15, H-21, and H-23. Compound **3** (17β-hydroxy-14,15-deoxy-gedunin) has not yet been isolated.

**Figure 4:**
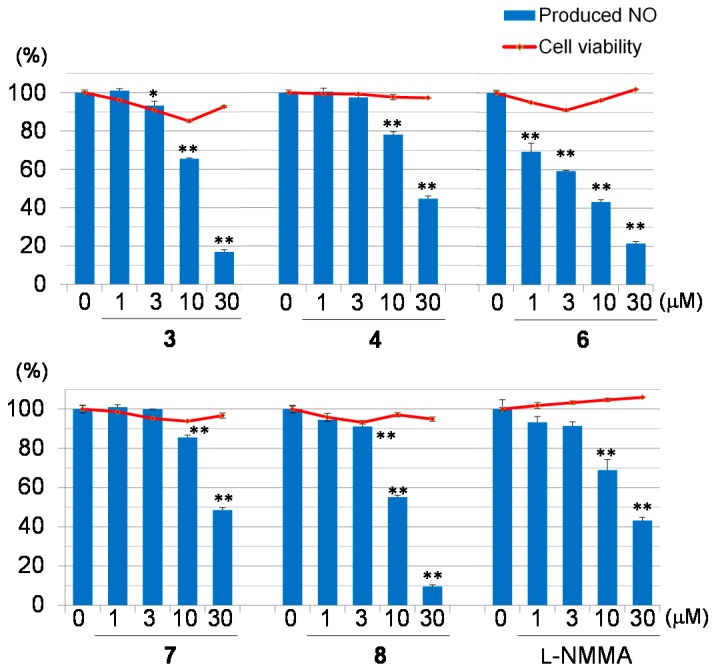
Inhibitory activities on NO production and cytotoxicities of Compounds **3**, **4**, **6**–**8** and L-NMMA. Each value represents the mean ± the standard error (S.E.) of four determinations. Significant differences from the vehicle control (0 μM) group shown as *: *p* < 0.05 and **: *p* < 0.01 in the NO inhibitory assay.

Macrophages may be a potential therapeutic target for inflammatory diseases [20]. Activated macrophages release pro-inflammatory mediators, such as NO, reactive oxygen species, interleukin-1 beta, tumor necrosis factor-alpha, and other inflammatory mediators, which play important roles in biological defense. However, the overexpression of these mediators has been implicated in diseases such as osteoarthritis, rheumatoid arthritis, and diabetes because the increased production of pro-inflammatory mediators has been shown to induce severe or chronic inflammation [21]. Eight limonoids, and L-NMMA, an inducible nitric oxide synthase (iNOS) inhibitor, were evaluated for their inhibitory effects on NO production (Figure 4). All tested compounds did not exhibit cytotoxicity (Cell viability 92.7%–100.4% at 30 μM). Of these, Compounds **3**, **6**, and **8** exhibited stronger inhibitory activity on NO production (IC_50_ **3**: 13.7 μM; **6**: 4.9 μM; **8**: 10.8) than L-NMMA (IC_50_ 23.9 μM). On the other hand, Compounds **4** and **7** showed moderate activity on NO production (IC_50_ **4**: 25.5 μM; **7**: 28.9 μM).

## 3. Experimental

### 3.1. General Experimental Procedures

Melting points were determined on a Yanagimoto micro-melting point apparatus and were uncorrected. Optical rotations were measured with a JASCO DIP-1000 digital polarimeter. IR spectra were recorded on a PerkineElmer 1720X FTIR spectrophotometer (Perkin-Elmer Inc., Wellesley, MA, USA). UV spectra were measured on a HITACHI U-2000 spectrometer using EtOH as a solvent. ^1^H- and ^13^C-NMR spectra were obtained on an Agilent vnmrs 600 spectrometer (Agilent Technologies, Santa Clara, CA, USA) with standard pulse sequences, operating at 600 and 150 MHz, respectively. CDCl_3_ was used as the solvent and TMS as the internal standard.

FABMS were recorded on a JEOL JMS-7000 mass spectrometer (JEOL, Tokyo, Japan). Column chromatography was performed over silica gel (70–230 mesh; Merck, Darmstadt, Germany), while medium pressure liquid chromatography (MPLC) was conducted with silica gel (230–400 mesh, Merck). HPLC was carried out using an ODS column [Cosmosil 5C_18_-MS column (Nacalai Tesque, Inc., Kyoto, Japan) (25 cm × 20 mm i.d.)] and a UV detector (220 nm) with 70% MeOH (isocratic) at a flow rate 4.0 mL/min. Injector fitted with a 100 μL loop. Fractions obtained from column chromatography were monitored by TLC (silica gel 60 F_254_; Merck).

### 3.2. Isolation of Compounds ***1**–**3***

Preliminary silica gel column chromatography was performed to separate the seed oil (1.1 kg) of *Carapa guianensis* A_UBLET_ into 8 fractions: Fraction A (Fractions 1–76, 900 g) was eluted with CHCl_3_, B (Fractions 77–110, 12.0 g) with CHCl_3_, C (Fractions 111–125, 21.0 g) with CHCl_3_/EtOAc = 5:1, D (Fractions 126–155, 10.9 g) with CHCl_3_/EtOAc = 5:1, E (Fractions 156–170, 1.4 g) with CHCl_3_/EtOAc = 2:1, F (Fractions 171–180, 2.4 g) with EtOAc, G (Fractions 181–195, 2.9 g) with EtOAc, and H (Fractions 196–208, 0.7 g) with EtOAc/MeOH = 5:1. Fraction E (1.4 g) was rechromatographed on a silica gel (70–230 mesh, 100 g) column using *n*-hexane/EtOAc = 1:1 to yield Residue E7 (426 mg). Residue E7 (426 mg) was rechromatographed on a silica gel (70–230 mesh, 100 g) column using *n*-hexane/EtOAc = 2:1 to yield Residues E11 (125 mg), E12 (33 mg), and E13 (43 mg). Residue E11 was separated by HPLC (ODS, 70% MeOH) to yield Compounds **7** and **8** (1.5 mg and 13.2 mg). Residue E12 was separated by HPLC (ODS, 65% CH_3_CN) to yield **4** (2.8 mg). Residue E13 was separated by HPLC (ODS, 70% MeOH) to yield **5** (1.5 mg). Fraction F (2.4 g) was rechromatographed on a silica gel (70–230 mesh, 120 g) column using *n*-hexane/EtOAc = 1:1 to yield Residues F1 (1.2 g) and F2 0.5 g). Residue F1 was rechromatographed on a silica gel (70–230 mesh, 600 g) column using *n*-hexane/EtOAc = 2:1 to yield Residue F2 (Fractions 88–101, 123 mg). Residue F2 (123 mg) was rechromatographed on a silica gel (230–400 mesh, 10 g) column using *n*-hexane/EtOAc = 2:1 to yield Residue F3 (71.0 mg). Residue C7 (71.0 mg) was separated by HPLC (ODS, 70% MeOH) to yield **6** (2.9 mg). Residue F2 (0.5 g) was rechromatographed on a silica gel (70–230 mesh, 10 g) column using *n*-hexane/EtOAc = 2:1 to yield Residue F4 (Fractions 33–50, 54.2 mg). Residue F4 was separated by HPLC (ODS, 50% CH_3_CN) to yield Carapanosin A (**1**) (3.4 mg), B (**2**) (2.9 mg), and C (**3**) (2.7 mg).

Carapanosin A (**1**): Colorless amorphous solid; m.p. 140–142 °C; ${\left[\alpha \right]}_{\text{D}}^{22}$ −74.6° (*c* 0.32, CHCl_3_); UV (EtOH) λ_max_ (log ε): 208 (3.52), 235.5 (3.54); IR (cm^−1^, KBr): 3647, 1751, 1700, 1652; FAB-MS *m*/*z* (rel.int.): 731 [M + H]^+^ (100), 671 (12), 95 (17); HR-FAB-MS *m*/*z* 731.2551 [M + H]^+^ (C_36_H_43_O_16_, calcd. 731.2551).

Carapanosin B (**2**): Colorless amorphous. ${\left[\alpha \right]}_{\text{D}}^{20}$ 64.0° (*c* 0.05, EtOH); UV (EtOH) λ_max_ (log ε): 237.5, 213 (log *ε* 3.62, 3.84); IR (cm^−1^, KBr): 3566, 1734, 1663, 1039. FAB-MS *m*/*z* (rel.int.): 773 [M + H]^+^ (49), 715 (65), 699 (77), 43 (100); HR-FAB-MS *m*/*z* 773.2659 [M + H]^+^ (C_38_H_45_O_17_, calcd. 773.2657).

Carapanosin C (**3**): Colorless crystal; m.p. 236–239 °C; ${\left[\alpha \right]}_{\text{D}}^{22}$ +80.5° (*c* 0.13, EtOH); UV (EtOH) λ_max_ (log ε): 238.5 (log ε 3.74); IR (cm^−1^, KBr): 3566 (OH), 1734, 1699, 1668, 1240, 1171; FAB-MS *m*/*z* (rel.int.): 505 [M + Na]^+^ (50), 483 [M + H]^+^ (77), 465 (23), 423 (52), 405 (14), 328 (25), 176 (37), 95 (100); HR-FAB-MS *m*/*z*: 483.2388 [M + H]^+^ (C_28_H_35_O_7,_ calcd. for 483.2383).

### 3.3. Cell Cultures

RAW264.7 cells (mouse macrophages) (obtained from DS Pharma Biomedical Co., Ltd. (Osaka, Japan)) were grown in DMEM. The medium was supplemented with 10% FBS and antibiotics (100 units/mL penicillin and 100 μg/mL streptomycin). The cells were incubated at 37 °C in a 5% CO_2_ humidified incubator.

### 3.4. Determination of RAW264.7 Cell Proliferation

RAW264.7 cell proliferation was examined in accordance with a method reported previously [22]. Briefly, RAW264.7 cells (5 × 10^4^ cells in 100 μL) were seeded onto a 96-well microplate, and incubated for 24 h. DMEM containing test samples (100 μL total volume, a final concentration of 30, 10, 3, or 1 μM) dissolved in DMSO (final concentration of 0.2%) was added. After treatment for 24 h, MTT solution was added. After a 3 h incubation, 20% sodium dodecyl sulfate in 0.1 M HCl was added to dissolve the formazan produced in the cells. The absorbance of each well was read at 570 nm using a microplate reader. The optical density of vehicle control cells was assumed to be 100%. Values are expressed as the mean ± standard error of the mean (S.E.M.). One-way ANOVA, followed by Dunnett’s test, was used for statistical analysis. Significant differences from the vehicle control (0 μM) group shown as *: *p* < 0.05 and **: *p* < 0.01.

## 4. Conclusions

Two new phragmalin-type limonoids, Carapanosins A (**1**) and B (**2**) as well as a gedunin-type limonoid, Carapanosin C (**3**) were isolated from the seeds of *Carapa guianensis*. Their structures were elucidated by spectroscopic analyses. In the NO inhibitory assay, Compounds **3**, **6**, and **8** exhibited similar NO inhibitory activities (IC_50_ **3**: 13.7 μM; **6**: 4.9 μM; **8**: 10.8 μM) to L-NMMA (IC_50_ 23.9 μM) without cytotoxicity. These results suggest that Compounds **3**, **6**, and **8** have potential as anti-inflammatory disease agents.

## Figures and Tables

**Table 1:** ^1^H-NMR and ^13^C-NMR Data of Compounds **1** and **2**.

Position		1		2		
		^1^H ^a^ (*J*, Hz)	^13^C ^b^	^1^H ^a^ (*J*, Hz)	^13^C ^b^	HMBC
1			84.5 (s)		84.1 (s)	
2			78.4 (s)		83.4 (s)	
3		4.57 s	83.8 (d)	5.19 s	85.3 (d)	4, 5
4			43.9 (s)		44.5 (d)	
5		2.94 d (10.9)	44.2 (d)	2.47 brd	44.7 (d)	4, 9, 10, 29
6	A	4.09 dd (12.1, 10.9)	71.4 (d)	6.31 brd	71.2 (d)	4, 5, 10
	B					
7			173.6 (s)		169.2 (s)	
8			83.0 (s)		83.5 (s)	
9			85.2 (s)		86.1 (s)	
10			48.7 (s)		48.8 (s)	
11	α	2.02 dd (14.7, 13.5)	31.9 (t)	2.00 t (14.1)	32.4 (t)	9, 10, 12, 13
	β	3.21 dd (14.7, 4.2)		2.35 dd (14.1, 4.1)		2, 8, 9, 12
12	α		68.8 (d)		68.5 (d)	17, 18
	β	4.99 dd (13.5, 4.2)		4.94 dd (14.1, 4.1)		
13			42.1 (s)		42.9 (s)	
14			162.7 (s)		152.6 (s)	
15		6.06 s	122.1 (d)	6.62 s	124.2 (d)	8, 14, 16,
16			159.6 (s)		163.4 (s)	
17		5.29 s	78.7 (d)	5.91 s	78.9 (d)	12, 13, 14, 18, 20, 22, 23
18		1.47 s	14.8 (q)	1.59 s	14.4 (q)	12, 13, 14, 17
19		1.34 s	14.8 (q)	1.31 s	16.4 (q)	1, 5, 9, 10
20			121.7 (s)		121.0 (s	
21		7.55 brs	141.7 (d)	7.45 brs	142.1 (d)	20, 22, 23
22		6.54 dd (1.7, 0.6)	110.2 (d)	6.56 dd (2.0, 1.2)	110.2 (d)	21, 23
23		7.42 t (1.7)	143.2 (d)	7.40 t (1.2)	143.0 (d)	20, 21, 22
28		0.89 s	15.7 (q)	0.92 s	15.3 (q)	3, 4, 5, 29
29	*pro-S*	1.75 d (10.8)	39.9 (t)	1.75 d (11.1)	40.8 (t)	1, 2, 3, 8
	*pro-R*	2.05 d (10.8)		2.23 d (11.1)		
30		5.71 s	68.9 (d)	5.35 s	74.1 (d)	1, 2, 8, 9
31			119.7 (s)		119.6 (s)	
32		1.68 s	21.0 (q)	1.69 s	16.4 (q)	31
1′			171.1 (s)		169.0 (s)	
2′		2.05 s	20.8 (q)	2.08 s	21.7 (q)	1′
1′′		3.78 s	52.5 (q)		171.7 (s)	7
2′′				2.20 s	20.9 (q)	7
1′′′′			172.3 (s)	3.74	53.3 (q)	
2′′′′		1.58 s	20.1 (q)			1′′′
1′′′′			174.0 (s)		170.4 (s)	
2′′′′	A	2.31 dq (10.5, 7.4)	27.8 (t)	1.55 s	19.8 (q)	1′′′′, 3′′′′
	B	2.42 m				
3′′′′		1.10 t (7.4)	8.9 (q)			1′′′′, 2′′′′
1′′′′′					173.9 (s)	
2′′′′′				2.43 dq (10.6, 7.3)	28.1 (t)	1′′′′′, 3′′′′′
				2.50 m		
3′′′′′				1.16 t (7.3)	8.9 (q)	1′′′′′, 2′′′′′
1-OH						
2-OH		3.65 s				

^a^ Measured at 600 MHz in CDCl_3_. ^b^ Measured at 150 MHz in CDCl_3_.

**Table 2:** ^1^H (600 MHz) and ^13^C (150 MHz) NMR spectroscopic data for Compound **3**.

Position		3		Position	3	
		^1^H ^a^ (*J*, Hz)	^13^C ^b^		^1^H ^a^ (*J*, Hz)	^13^C ^b^
1		7.06 d (10.3)	156.4 (d)	14		170.3 (s)
2		5.87 d (10.3)	125.8 (d)	15	5.64 s	111.0 (d)
3			203.8 (s)	16		163.4 (s)
4			43.7 (s)	17		104.0 (s)
5		2.16 dd (12.6, 4.1)	45.5 (d)	18	1.16 s	23.3 (q)
6	α	1.97 m	23.0 (t)	19	1.25 s	18.8 (q)
	β	1.99 m		20		125.0 (s)
7		5.25 t (2.9)	73.2 (d)	21	7.58 brs	142.9 (d)
8			44.4 (s)	22	6.48 dd (1.8, 0.9)	125.0 (d)
9		2.20 dd (11.4, 8.5)	37.2 (d)	23	7.43 t (1.8)	141.6 (d)
10			40.4 (s)	28	1.08 s	26.8 (q)
11	α	2.00 m	15.1 (t)	29	1.09 s	21.1 (q)
	β	1.86 ddd (14.1, 11.4, 1.7)		30	1.36 s	24.1 (q)
12	α	2.30 dt (14.1, 9,9)	23.2 (t)	1′		169.6 (s)
	β	1.60 m		2′	1.98 s	20.7 (q)
13			42.0 (s)			

^a^ Measured at 600 MHz in CDCl_3_. ^b^ Measured at 150 MHz in CDCl_3_. Assignment are based on HMBC spectrum.
